# Starch Components, Starch Properties and Appearance Quality of Opaque Kernels from Rice Mutants

**DOI:** 10.3390/molecules24244580

**Published:** 2019-12-13

**Authors:** Shun Zhang, Zheng Li, Lingshang Lin, Long Zhang, Cunxu Wei

**Affiliations:** 1 Key Laboratory of Crop Genetics and Physiology of Jiangsu Province/Jiangsu Key Laboratory of Crop Genomics and Molecular Breeding, Yangzhou University, Yangzhou 225009, Chinayzulizheng@163.com (Z.L.); 18252713442@163.com (L.L.); zhanglong@yzu.edu.cn (L.Z.); 2 Co-Innovation Center for Modern Production Technology of Grain Crops of Jiangsu Province/Joint International Research Laboratory of Agriculture & Agri-Product Safety of the Ministry of Education, Yangzhou University, Yangzhou 225009, China

**Keywords:** rice mutant, starch component, physicochemical properties, appearance quality

## Abstract

Rice mutants with altered starch components and properties are important genetic resources in rice breeding programmes. In this study, 44 mutants with altered starch components were screened from 135 rice mutants with opaque kernels using a starch–iodine absorption spectrum method, and nine mutants from them were further selected for investigating their starch properties and kernel appearance quality. The results showed that the iodine absorption spectrum parameters, OD620, OD620/550, and λmax, could reflect the changes of starch components in rice mutants, and had significantly positive relationships with amylose content and negative relationships with the proportion of short branch-chains of amylopectin. The endosperm starches from nine mutants all showed A-type crystalline structure and similar short-range ordered structure, but had different relative crystallinities. The changes of starch components in mutants not only resulted in the different gelatinization properties of starch but also changed the appearance quality of brown rice kernels. This study provided abundant genetic plants for studying the molecular mechanism of starch synthesis and the quality regulation of rice kernels.

## 1. Introduction

Rice is one of the most important crops, and provides the staple food for over half the world’s population. Starch is the main component of rice endosperm, and its properties greatly influence the eating and cooking qualities of rice and its processing method in industrial use [1,2,3]. Therefore, the cultivation of rice varieties with different starch properties is one of the important goals in rice breeding. Starch mainly consists of amylose and amylopectin, and the starch components including amylose content (AC) and amylopectin structure determine starch properties [4,5]. According to the AC, rice varieties are usually classified into waxy (0–2%), very low (3–9%), low (10–19%), intermediate (20–25%), and high (>25%) amylose classes [6]. The AC plays a crucial role in determining the cooking quality of rice; for example, the cooked rice with low AC exhibits the advantages of smoothness, being refreshing, elasticity, and having a low degree of retrogradation; and cooked rice with high AC is dry and hard [7,8,9]. In addition, the amylopectin structure also influences the properties of starch. The rice starch with long branch-chains of amylopectin and B-type crystalline contains high resistant starch, and has health benefits [4,5,10]. Therefore, it is important to cultivate rice varieties with different ACs and amylopectin structures.

In recent years, some novel rice materials with different qualities have been cultivated through traditional cross breeding, chemical or physical mutagenesis breeding, and advanced biotechnological approaches, including transfer DNA or transposon insertion and RNA interference [10,11,12,13,14,15,16,17,18]. These mutants have significantly different starch properties and expand the uses of rice kernels not only as special food for human health but also as important materials for industry product. Most importantly, these mutants are valuable genetic materials for studying the molecular mechanism of starch synthesis and quality regulation of rice kernels. The mechanism’s understanding could accelerate the cultivation of novel rice varieties with improved rice quality [11]. In various kinds of seed mutants, an important phenotype character is that mature kernels are usually opaque and have waxy or floury endosperm. For example, the mutation of genes responsible for protein synthesis, storage, and transport result in floury endosperm [12,13]. A mutant with increased aleurone thickness has floury endosperm [14]. Mutating starch synthesis related genes produces floury kernels [15,16,17]. In addition, the mutation of transcript factor regulating starch and storage protein synthesis genes also results in opaque kernels [18]. The opaque phenotype of kernels is easily detected from rice mutants; however, not all opaque kernels have the changes in starch components. Therefore, it is very necessary, and in theory, possible to rapidly screen mutants with altered starch components and properties from rice mutants with opaque kernels.

In this study, we screened mutants with altered starch components from rice mutants with opaque kernels. Their starch properties and kernel appearance qualities were investigated. This study should provide valuable genetic materials to study the molecular mechanism of starch synthesis, and provide important information for rice quality breeding.

## 2. Results and Discussion

### 2.1. Screening Mutants with Altered Starch Components

**Figure 1:**
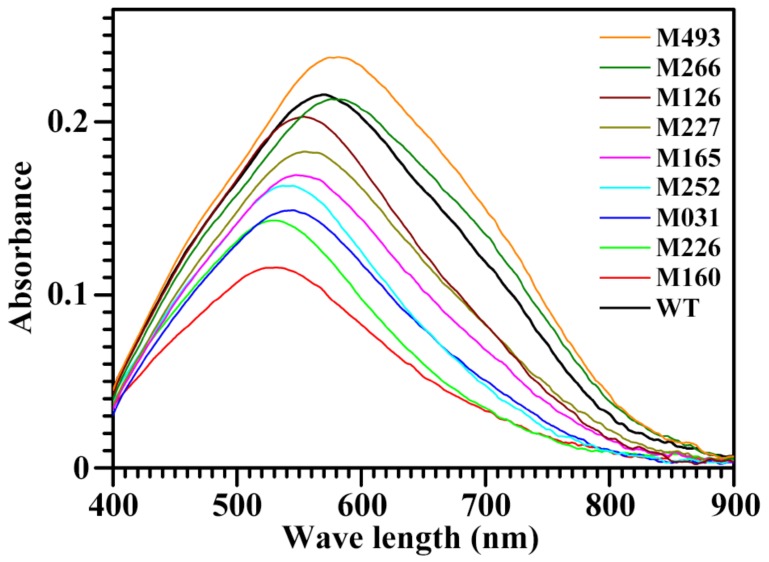
Iodine absorbance spectra of starches from brown rice kernels.

Starch contains two main components: amylose and amylopectin. Their contents can be simply detected through measuring their iodine absorption spectra [19]. The iodine absorption spectra of starches from some mutants are shown in Figure 1. The iodine absorption spectrum parameters of starches from 135 rice mutants which are opaque are summarized in Table S1. The λmax is the maximum absorption wavelength and is related to the polymerization degree and average chain length of amylose and amylopectin. The OD620 is usually used to assess the AC of starch. The OD620/OD550 can reflect the relative content of longer chain segments in starch [19]. Therefore, the changes of starch components can be detected through analysis of OD620, OD620/OD550, and λmax.

Except the starch-iodine colorimetry, the methods for measuring starch components are concanavalin A precipitation, gel permeation chromatograph (GPC), size-exclusion chromatography, near-infrared analysis, and differential scanning calorimetry (DSC) analysis [4,19,20]. These methods contain labour-intensive and time-consuming processes and are not suitable for screening mutants with altered starch components from a large number of germplasm samples. In order to rapidly screen mutants with altered starch components from a great quantity of mutants, Agasimani et al. [20] established a simple and rapid single kernel screening method. The method involves transversely cutting the rice transparent kernels in the middle region and dipping the cut end in an optimized iodine solution to estimate the AC in a rice kernel. The time taken for deep blue colouration by the cut end of the kernel after dipping in iodine solution is positively correlated to the AC. However, it is very difficult to judge the colouration of deep blue. In addition, the method is not suitable for kernels with slightly altered AC or with floury endosperm. However, the rice mutants with altered starch components usually have floury endosperm; there is no way to screen the floury mutants with altered starch components using the method of Agasimani et al. [20]. In the present study, the starch components were seen as changes when the differences of OD620, OD620/OD550, and λmax between mutants and wild type (WT) rice were over 0.02, 0.04, and 7 nm, respectively. According to this standard, 44 rice mutants rapidly screened from 135 rice mutants were found to have the changes of starch components (Table S1). According to the OD620, we further selected nine mutants with OD620 ranging from 0.071 to 0.219 from the 44 mutants to represent the different extents of altered starch components. The iodine absorbance spectra and spectrum parameters of the selected nine mutant starches are summarized in Figure 1 and Table 1. The molecular components and properties of the nine mutant starches were further investigated in the present study.

### 2.2. Starch Components in Brown Kernels of Rice Mutants

The AC in isolated starch was measured using Megazyme amylose/amylopectin assay kit (Table 2). According to the principle of the kit, the amylose and amylopectin in isolated starch are dissolved completely and quantified. The AC is the percentage of the amylose to both amylose and amylopectin. Therefore, its value is not affected by the starch purity and usually called true AC. The starch from WT rice had 13.3% AC, and starches from nine selected rice mutants had ACs ranging from 2.2% to 14.3%. Many opaque mutants have low ACs, especially for the one with the mutated *granule-bound starch synthase I gene* [21,22].

**Figure 2:**
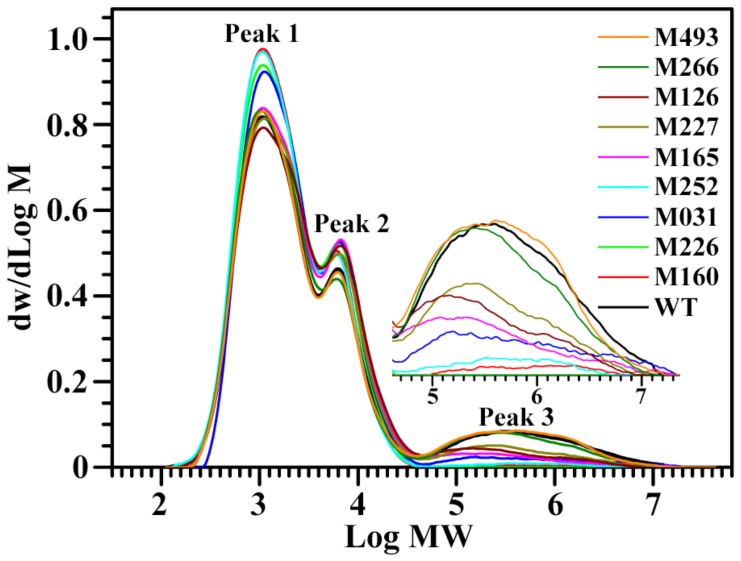
GPC profiles of isoamylase-debranched starches from brown rice kernels.

In order to further confirm the component changes of starch, the isoamylase-debranched starch was analysed using GPC (Figure 2). The three peaks, Peak 1, Peak 2, and Peak 3, of GPC profiles reflect the amylopectin short branch-chains, amylopectin long branch-chains, and amylose molecules, respectively. Their area percentages can assess the contents of starch components, and the area ratio of amylopectin’s short to long branch-chains can reflect the branching degree of amylopectin [23]. In the present study, starch from WT contained 63.9% amylopectin short branch-chains, 23.3% amylopectin long branch-chains, and 12.8% amylose, and had a 2.75 amylopectin branching degree. However, starches from mutants had amylopectin short branch-chains in proportions from 64.7% to 75.8%, amylopectin long branch-chains from 20.8% to 29.1%, amylose from 0 to 13.0%, and amylopectin branching degrees from 2.39 to 3.29 (Table 2). The amylose is synthesized by granule-bound starch synthase I, and the amylopectin is synthesized by the combined actions of soluble starch synthase, starch branching enzyme, and starch debranching enzyme. Mutants with a mutation or expression change of starch-synthesis-related enzyme genes can alter starch components [24,25].

### 2.3. Relationships between Iodine Absorption Spectrum Parameters and Starch Components

In order to reveal whether the changes of iodine absorption spectrum parameters can reflect the changes of starch components, their relationships were analysed (Table 3). The OD620, OD550, OD620/OD550, and λmax were significantly positively correlated to AC and negatively correlated to the content of amylopectin short branch-chains (*p* < 0.01). Similar results have been reported in the previous paper [19]. The present study indicated that the mutants with changed starch components could be rapidly detected. Though the Lugol-based coloration assay is fast and reliable in detecting the waxy mutants, it is difficult to detect the non-waxy mutants with changed AC and amylopectin structures using a Lugol-based coloration assay. Therefore, the iodine absorption spectrum method had special potential and usefulness in rapidly screening the non-waxy mutants with changed starch components.

**Figure 3:**
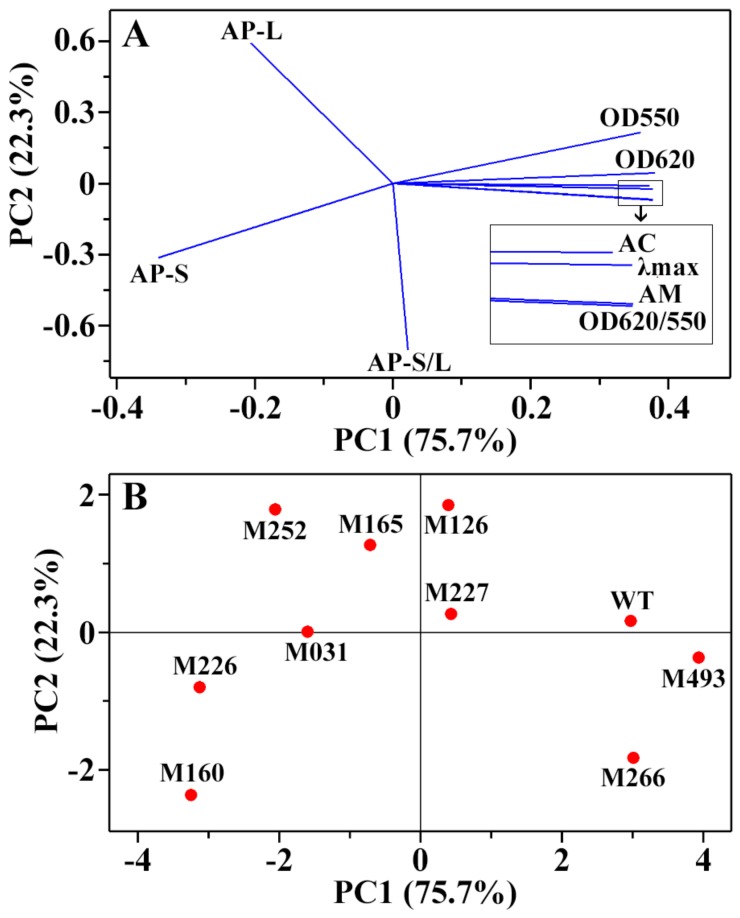
Loading (**A**) and score plots (**B**) of principal component analysis based on iodine absorption spectrum parameters and starch components.

Principal component analysis (PCA) is a widely used method to determine the relationships of variables that contribute to differences between physicochemical properties or samples. The iodine absorption spectrum parameters and starch components of ten rice materials were subjected to PCA, and the results are present in Figure 3. The PC1 and PC2 explained 75.7% and 22.3% of the overall variation, respectively. For the loading plot of PCA, the properties with curves close to each other on the plot are positively correlated while those with curves in opposite directions are significantly negatively correlated. The loading plot of iodine absorption spectrum parameters and starch components showed that OD550, OD620, AC, λmax, AM, and OD620/550 were highly positive correlated, and they were negatively correlated to the amylopectin short branch-chains (Figure 3A). The score plot of PCA provides an overview of the similarities and differences between the starches from different rice materials. The distance between the locations of any two starches can reflect the degree of the difference and similarity between them. The score plot of PCA showed that starches from different rice mutants had some differences in their starch components (Figure 3B).

### 2.4. Crystalline Structure of Starches from Rice Mutants

**Figure 4:**
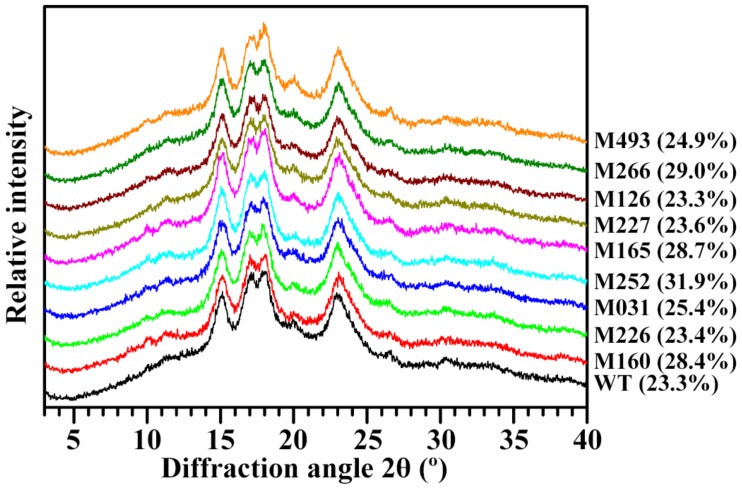
XRD patterns of starches from brown rice kernels. The relative crystallinity of starch is given in the parentheses after the sample name.

The X-ray diffraction (XRD) patterns of starches are shown in Figure 4. Native starches from different plant sources can be classified into A-, B- or C-type according to their XRD patterns. The starches from normal cereal endosperm have A-type crystallinity; tuber starches show B-type crystallinity; and rhizome and legume starches have C-type crystallinity [26,27]. In the present study, starches from WT rice and its derived nine mutants all had typical A-type XRD patterns with strong diffraction peaks at 15° and 23° 2θ, and an unresolved doublet at 17° and 18° 2θ (Figure 4). The peak at 20° 2θ is the structure of V-type crystallinity and is usually related with amylose-lipid complex [28]. The intensity of peak at 20° 2θ had no significant difference between WT rice and mutant lines except that the M493 line had a slightly high peak intensity (Figure 4). This result might indicate that lipid content in starch was similar between mutant lines and WT rice. Usually, the mutation of starch branching enzyme gene can change the amylopectin structure and increase AC, leading to the change of crystalline structure from A-type to B-type or C-type. However, mutating *granule-bound starch synthase I* or *soluble starch synthase* does not change to the crystalline structure of starch, though starch components change significantly [29]. The relative crystallinity ranged from 23.3% to 31.9% among WT rice and nine mutants (Figure 4). Usually, the relative crystallinity is significantly negatively correlated with AC and positively correlated with amylopectin short branch-chains [26,27,30]. However, in the present study, Pearson correlation coefficients (data not shown) showed that there was no significant relationship between relative crystallinity and starch components, indicating that some non-starch components in starch granules might influence the relative crystallinity.

### 2.5. Short-Range Ordered Structure of Starches from Rice Mutants

**Figure 5:**
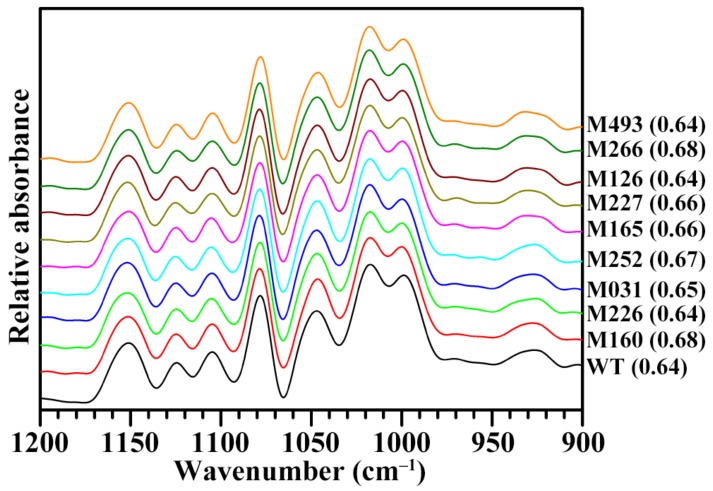
FTIR spectra of starches from brown rice kernels. The short-range ordered degree of starch (1045/1022 cm^−1^) is given in the parentheses after sample name.

The branch-chains of amylopectin form two types of helices in starch granules. The helices that are packed in short-range order are defined as the double helical order, and the helices that are packed in long-range order are related to the packing of double helices forming crystallinity [31]. The short-range ordered structure of amylopectin can be detected by infrared. The attenuated total reflectance-Fourier transform infrared (ATR-FTIR) is usually used to detect the short-range ordered structure of amylopectin in the external region of starch granule. The ATR-FTIR spectra of starches are shown in Figure 5. For FTIR spectra of starches, the bands at 1045 and 1022 cm^−1^ have been linked with order/crystallinity and amorphous regions in starch, respectively. The ratio of absorbance 1045 to 1022 cm^−1^ (1045/1022 cm^−1^) is usually used to assess the ordered degree of starch [32]. The FTIR spectra of starches did not have significant differences among WT rice and nine mutants, indicating a similar short-range order structure of amylopectin in the external region of starch granules (Figure 5). The present results agreed with the previous report that the normal maize starch and waxy maize starch have similar ordered degree [32].

### 2.6. Thermal Properties of Starches from Rice Mutants

**Figure 6:**
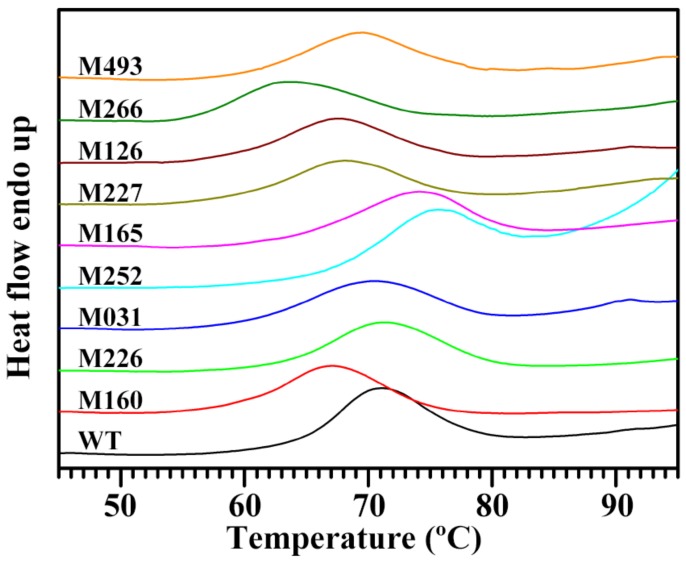
DSC thermal curves of starches from brown rice kernels.

The thermal properties of starch are important functional properties and determine the applications of starch. The thermal properties of starches are usually determined by DSC, which measures the change of heat involved in starch gelatinization. The gelatinization results in the disruption of double helical and crystalline structure of amylopectin in starch granules. The DSC thermal curves of starches from rice mutants are shown in Figure 6, and their thermal parameters are summarized in Table 4. The starches from different mutants had significantly different gelatinization temperatures and enthalpies. Though the M252 starch had a low AC (1.3%), it exhibited the highest gelatinization temperature and the lowest gelatinization temperature range and enthalpy. The M266 starch contained the medium AC (11.9%) but had the lowest gelatinization temperature (Table 2 and Table 4). From the point of the effect of AC on thermal properties, contradictory results have been reported [30,33,34,35,36,37]. For instance, some papers report that AC is positively correlated with gelatinization temperature [30,33,34], while other references show that waxy starch has higher gelatinization temperature than normal starch [35,36,37]. The conflicting conclusion is ascribed to the different genetic backgrounds of the rice materials tested [37]. Even in a common rice genetic background with different *Wx* alleles and ACs, the starch with lower AC has a higher gelatinization temperature. This is due to the fact that the lower amylose contributes to the higher relative crystallinity of starch, needing a higher temperature to disrupt it [37]. In fact, the thermal properties of starch are influenced by many factors, such as granule size, AC, amylopectin structure, crystalline structure, and some non-starch components [38]. The change of amylopectin structure affects the thermal properties more than the AC change [35,37]. Among the ten starches, the M252 and M266 starches had the highest (29.1%) and the lowest (20.8%) amylopectin long branch-chain contents, respectively (Table 2), and might accordingly need the highest and the lowest temperatures to dissociate them completely (Table 4). The results were in agreement with the previous report [39]. In the present study, the different thermal properties of starches from different mutants might be due to amylopectin’s molecular structure and AC jointly playing important roles in determining starch gelatinization. In a future work, which gene mutation influenced the starch components and their relations to thermal properties need to be fully clarified.

### 2.7. Appearance Quality of Brown Rice Kernels

**Figure 7:**
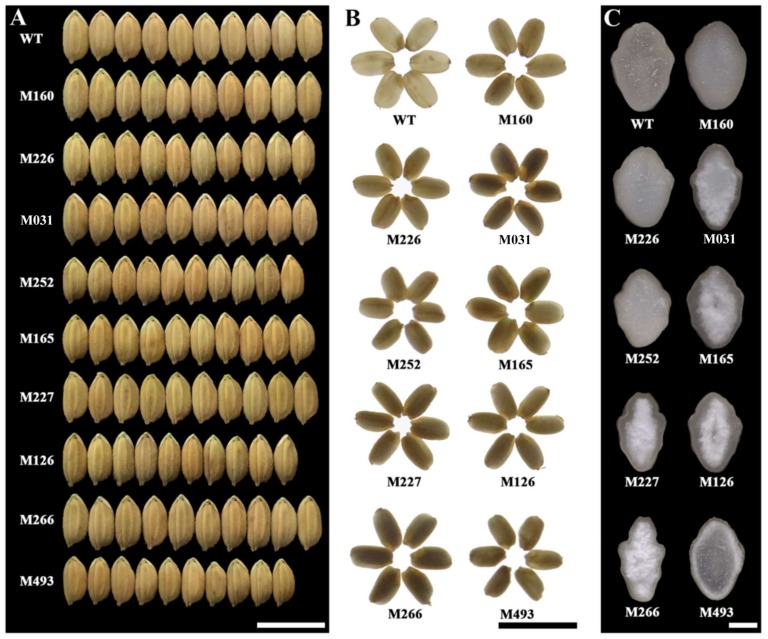
Morphologies of grains (**A**), brown rice kernels (**B**), and cross sections from the mid-regions of brown rice kernels. Scale bar = 1 cm for (**A**,**B**) and 1 mm for (**C**).

The unhulled mature grains are shown in Figure 7A. No significant differences were observed except for the slight change of grain size. The brown rice kernels were observed under transmitted light. The WT kernels were transparent, but the mutant kernels showed an opaque phenotype (Figure 7B). In order to reveal the reasons for poor transparency of kernels from mutants, the cross fractured planes of brown rice kernels were observed under stereoscopic microscope (Figure 7C). The M160, M226, and M252 kernels were waxy and opaque, the other mutants had floury endosperms. It has been widely reported that the waxy rice kernels with ACs below 2% result from a deficiency of granule-bound starch synthase I and exhibit a typically waxy phenotype [40]. Recently, Zhang et al. [41] reported that the waxy phenotype of a dry kernel is due to the cavities in the centre of starch granules. The kernels with altered starch components in non-waxy rice mutants usually exhibit floury phenotypes, due to the fact that the synthesis of starch is influenced and starch granules are loosely packed in endosperm cells [42]. In the present study, the morphology observations of brown kernels of rice mutants were in agreement with the results of starch component (Table 2). The M160, M226, and M252 lines had ACs below 2.0%, and exhibited waxy kernels. The M031, M165, M227, M126, M266, and M493 lines had AC changes from 4.0% to 13.0% and exhibited floury kernels. The dehulled brown rice kernels were measured for their length, width, thickness, and weight. Some mutant kernels had significantly lower kernel length, width, thickness, and weight values than WT kernels, but some mutant kernels had similar kernel shapes and weights to WT kernels (Table 5).

Compared with floury endosperm mutants with slightly changed starch components due to the mutation of storage protein synthesis or aleurone cell development [12,13,14], the newly screened non-waxy floury endosperm M031, M165, M227, M126, M266, and M493 lines had significantly changed starch components, starch properties, and appearance quality of kernel. Some rice floury endosperm materials resulting from the mutation or regulating expression of starch synthesis related genes have been reported; their starch components, starch properties and kernel quality significantly change [10,11,15,16,17,18]. However, it is very complicated to understand and regulate the starch synthesis and kernel quality, and more mutants are needed to reveal the regulation mechanism. Therefore, the newly selected non-waxy mutants with significantly changed starch components were valuable genetic materials for further investing the molecular mechanism of starch synthesis and the quality regulation of rice kernels.

## 3. Materials and Methods

### 3.1. Plant Materials

The rice mutants with opaque kernels were derived from a *japonica* rice (*Oryza sativa* L.) variety (WT), Kitaake, through ^60^Co gamma irradiation [43]. These mutants were homozygotes and self-pollinated for three generations. The WT rice and mutants were planted simultaneously in an experiment field of Yangzhou University, Yangzhou, China. The mature grains were dehulled, and the brown kernels were used as plant materials in this study.

### 3.2. Isolation of Starch from Brown Rice Kernels

Starch was isolated from brown rice kernels following the method of Lin et al. [19] with some modifications. Briefly, mature and dry kernels were extensively ground into powder with a mortar, and then were ground in deionized water. The starch-water slurry was filtered through 150 and 75 μm sieve, and centrifuged. The starch precipitation was washed 3 times using deionized water, and the top dirty materials were carefully removed. The starch was dehydrated two times using anhydrous ethanol, dried at 40 °C, and ground through 150 μm sieve. The samples were stored at 4 °C.

### 3.3. Determination of Starch–Iodine Absorption Spectrum

The iodine absorption spectrum of starch was measured exactly following the method of Lin et al. [19]. Briefly, starch was dissolved in dimethyl sulfoxide (DMSO) containing 10% 6 M urea, and coloured with I_2_/KI. The sample was scanned from 400 to 900 nm using a spectrophotometer (Ultrospec 6300 pro, Amersham Biosciences, Cambridge, UK).

### 3.4. Determination of AC in Starch

The AC in isolated starch was measured using an amylose/amylopectin assay kit (Megazyme, Bray, Ireland).

### 3.5. Determination of Molecular Weight Distribution of Starch

The molecular weight distribution of starch was analysed using GPC following the method of Lin et al. [19]. Briefly, starch was deproteinized first with protease and sodium bisulfite, then dissolved in hot water, and finally, debranched finally using isoamylase. The debranched starch was analysed using a GPC system (PL-GPC 220, Agilent Technologies UK Limited, Shropshire, UK) with three columns (PL110-6100, 6300, 6526) and a differential refractive index detector.

### 3.6. Analysis of Crystalline Structure of Starch

The crystalline structure of starch was measured using X-ray diffractometer (D8, Bruker, Karlsruhe, Germany) following the method of Lin et al. [4]. The test conditions included the voltage of 40 kV, electric current of 200 mA, step size of 0.02°, and scanning range from 4° to 40° 2θ.

### 3.7. Analysis of Short-Range Ordered Structure of Starch

The short-range ordered structure of starch was measured using an attenuated total reflectance–Fourier transform infrared (ATR–FTIR) spectrometer (7000, Varian, Santa Clara, CA, USA) following the method of Lin et al. [4]. The test conditions included the scanning range from 400 to 4000 cm^−1^, resolution of 4 cm^−1^, and scan times of 64. The original spectrum was corrected by a baseline from 1200 to 800 cm^−1^, and deconvoluted using a Resolutions Pro with resolution enhancement factor of 1.9 and peak half-width of 1.9 cm^−1^.

### 3.8. Determination of Thermal Properties of Starch

Five mg of starch and 15 μL deionized water were mixed and sealed in an aluminium pan. The sample was equilibrated at room temperature for 2 h, and then analysed using a differential scanning calorimeter (200-F3, Netzsch, Selb, Germany). The test conditions included a heating rate of 10 °C/min and a temperature range from 20 to 130 °C.

### 3.9. Measurement of Appearance Quality of Brown Rice Kernels

The hulled grains and dehulled brown rice kernels were photographed with a digital camera (IXUS 750, Canon, Tokyo, Japan). The cross section of brown rice kernel at the middle region was photographed using a Stereo Microscope (EZ4W, Leica, Wetzlar, Germany). The lengths, widths, and thicknesses of 30 brown rice kernels were measured using an electronic digital calliper (0–150, Shanghai Shenhan Measuring Tools Co., Shanghai, China). The 100 brown rice kernels were weighed and repeated three times.

### 3.10. Statistical Analysis

The data were analysed using the SPSS 16.0 Statistical Software Program, and the one-way analysis of variance was evaluated using Tukey’s test. The principle component analysis was carried out using Minitab Version 16.0 Software (IBM Company, Chicago, IL, USA).

## 4. Conclusions

The starch-iodine absorption spectrum parameters: OD620, OD620/550, and λmax, could reflect the component changes of starch. Forty-four rice mutants with ACs from 2.2% to 14.3% were rapidly screened from 135 rice mutants with opaque kernels according to the changes of starch-iodine absorption spectrum parameters. The screened mutants had significantly different starch molecular weight distributions but exhibited the same A-type crystallinity and similar short-range ordered structures. The mutants with different starch components had different thermal properties of starch, and exhibited significant differences in length, width, thickness, and weight of their brown rice kernels. The newly selected non-waxy floury mutants are valuable genetic materials for further investing the molecular mechanism of starch synthesis and the quality regulation of rice kernels.

## Figures and Tables

**Table 1:** Iodine absorption spectrum parameters of starches from brown rice kernels.

Rice Materials	OD620	OD550	OD620/OD550	λmax (nm)
WT	0.188 ± 0.004e	0.210 ± 0.009f	0.89 ± 0.02g	568.2 ± 1.0e
M160	0.071 ± 0.006a	0.112 ± 0.009a	0.63 ± 0.00b	529.8 ± 0.7a
M226	0.082 ± 0.009a	0.137 ± 0.015b	0.60 ± 0.00a	529.5 ± 1.8a
M031	0.102 ± 0.007b	0.148 ± 0.012bc	0.69 ± 0.01d	542.7 ± 2.5b
M252	0.107 ± 0.003b	0.162 ± 0.006cd	0.66 ± 0.01c	540.4 ± 0.5b
M165	0.127 ± 0.005c	0.169 ± 0.007cd	0.75 ± 0.01e	549.9 ± 0.3c
M227	0.145 ± 0.007d	0.182 ± 0.010de	0.80 ± 0.01f	557.6 ± 4.0d
M126	0.154 ± 0.003d	0.202 ± 0.003ef	0.76 ± 0.00e	552.8 ± 0.6cd
M266	0.195 ± 0.009e	0.202 ± 0.010ef	0.97 ± 0.01h	579.7 ± 1.9f
M493	0.219 ± 0.007f	0.224 ± 0.010f	0.98 ± 0.02h	579.5 ± 3.5f

Data are means ± SDs, *n* = 3. Values in the same column with different letters are significantly different.

**Table 2:** Components of starches from brown rice kernels.

Rice Materials	AC (%)	AP-S (%)	AP-L (%)	AM (%)	AP-S/L
WT	13.3 ± 0.2f	63.9 ± 0.5a	23.3 ± 0.5b	12.8 ± 0.0e	2.75 ± 0.08b
M160	2.2 ± 0.1a	75.8 ± 0.1e	23.1 ± 0.0b	1.1 ± 0.1a	3.29 ± 0.01d
M226	3.0 ± 0.2ab	74.6 ± 0.2e	25.4 ± 0.2c	0.0 ± 0.0a	2.94 ± 0.03c
M031	4.1 ± 0.4b	70.4 ± 0.3d	25.6 ± 0.4c	4.0 ± 0.1b	2.74 ± 0.05b
M252	3.2 ± 0.1ab	69.6 ± 0.6cd	29.1 ± 0.0e	1.3 ± 0.6a	2.39 ± 0.02a
M165	5.3 ± 0.4c	68.1 ± 0.1bc	27.4 ± 0.0d	4.5 ± 0.1b	2.48 ± 0.00a
M227	6.2 ± 0.6c	67.5 ± 0.7b	24.9 ± 0.3c	7.6 ± 1.0d	2.71 ± 0.00b
M126	8.2 ± 0.3d	66.4 ± 0.2b	27.7 ± 0.1d	5.9 ± 0.0c	2.40 ± 0.02a
M266	10.7 ± 0.5e	67.3 ± 0.9b	20.8 ± 0.5a	11.9 ± 0.4e	3.23 ± 0.13d
M493	14.3 ± 0.4f	64.7 ± 0.5a	22.4 ± 0.2b	13.0 ± 0.3e	2.89 ± 0.05bc

Data are means ± SDs, *n* = 3. Values in the same column with different letters are significantly different. AC, the amylose content in starch measured by amylose/amylopectin assay kit; AP, amylopectin; AP-S, short branch-chains of AP; AP-L, long branch-chains of AP; AP-S/L, ratio of AP-S and AP-L; AM, amylose. The AP-S, AP-L, and AM are measured through the GPC of isoamylase-debranched starch.

**Table 3:** Pearson correlation coefficients between iodine absorption parameters and starch components.

	OD620	OD550	OD620/550	λmax	AC	AP-S	AP-L	AM
OD550	0.964 ^**^							
OD620/550	0.975 ^**^	0.890 ^**^						
λmax	0.981 ^**^	0.913 ^**^	0.995 ^**^					
AC	0.967 ^**^	0.919 ^**^	0.938 ^**^	0.927 ^**^				
AP-S	−0.899 ^**^	−0.955 ^**^	−0.831 ^**^	−0.858 ^**^	−0.854 ^**^			
AP-L	−0.474	−0.246	−0.606	−0.549	−0.539	0.113		
AM	0.961 ^**^	0.885 ^**^	0.978 ^**^	0.969 ^**^	0.961 ^**^	−0.851 ^**^	−0.618	
AP-S/L	−0.003	−0.244	0.156	0.092	0.067	0.395	−0.864 ^**^	0.145

The abbreviations of AC, AP-S, AP-L, and AM are explained in Table 2. **p < 0.01.

**Table 4:** Thermal parameters of starches from brown rice kernels.

Rice Materials	To (°C)	Tp (°C)	Tc (°C)	Δ*T* (°C)	Δ*H* (J/g)
WT	64.4 ± 0.1e	71.0 ± 0.2e	78.1 ± 0.8c	13.8 ± 0.8a	10.9 ± 0.1d
M160	59.1 ± 0.1b	67.1 ± 0.1b	75.1 ± 0.5ab	16.0 ± 0.6b	9.7 ± 0.6bcd
M226	62.9 ± 0.1d	71.3 ± 0.1e	79.9 ± 0.3d	17.1 ± 0.4bc	10.4 ± 0.0cd
M031	60.2 ± 0.1c	70.7 ± 0.3e	79.7 ± 0.7d	19.5 ± 0.8d	9.3 ± 0.5abcd
M252	67.9 ± 0.2f	75.7 ± 0.2g	81.6 ± 0.6e	13.8 ± 0.8a	7.8 ± 0.3a
M165	63.6 ± 0.2d	74.2 ± 0.0f	81.8 ± 0.6e	18.3 ± 0.8cd	10.8 ± 1.0d
M227	59.2 ± 0.1b	68.3 ± 0.1c	77.1 ± 0.6c	17.9 ± 0.4bcd	8.8 ± 0.3abc
M126	59.5 ± 0.6bc	67.9 ± 0.4c	76.4 ± 0.1bc	16.9 ± 0.6bc	8.5 ± 0.8ab
M266	55.7 ± 0.1a	63.9 ± 0.3a	74.2 ± 0.1a	18.5 ± 0.1cd	8.6 ± 0.2abc
M493	60.2 ± 0.1c	69.5 ± 0.1d	78.0 ± 0.0c	17.8 ± 0.1bcd	8.6 ± 0.3abc

Data are means ± SDs, *n* = 3. Values in the same column with different letters are significantly different. To, gelatinization onset temperature; Tp, gelatinization peak temperature; Tc, gelatinization conclusion temperature; ΔT, gelatinization temperature range; ΔH, gelatinization enthalpy.

**Table 5:** Length, width, thickness, and weight values of brown rice kernels.

Rice Materials	Length (mm)	Width (mm)	Thickness (mm)	Weight (g/100 Kernels)
WT	5.11 ± 0.12d	3.02 ± 0.07d	2.10 ± 0.07ef	2.23 ± 0.01h
M160	4.77 ± 0.13b	2.92 ± 0.09bc	2.08 ± 0.04ef	2.01 ± 0.02f
M226	5.14 ± 0.16d	2.88 ± 0.11b	2.09 ± 0.06ef	2.22 ± 0.03h
M031	4.80 ± 0.11b	2.99 ± 0.09cd	2.11 ± 0.06f	2.11 ± 0.00g
M252	4.71 ± 0.06b	2.89 ± 0.08b	2.04 ± 0.05de	1.95 ± 0.01e
M165	4.72 ± 0.17b	2.88 ± 0.09b	1.94 ± 0.19bc	1.65 ± 0.02b
M227	4.95 ± 0.08c	2.96 ± 0.07bcd	1.98 ± 0.07cd	1.81 ± 0.01d
M126	4.94 ± 0.13c	2.77 ± 0.16a	1.93 ± 0.11bc	1.81 ± 0.01d
M266	5.28 ± 0.18e	2.95 ± 0.25cd	1.64 ± 0.10a	1.59 ± 0.00a
M493	4.59 ± 0.12a	2.75 ± 0.10a	1.89 ± 0.09b	1.69 ± 0.02c

Data are means ± SDs, *n* = 3. Values in the same column with different letters are significantly different.

## Supplementary Materials

The following are available online. Table S1: Iodine absorbance spectrum parameters of starches from brown rice kernels.
