# Supplementary material for: Starch Components, Starch Properties and Appearance Quality of Opaque Kernels from Rice Mutants
Source: Molecules. 2019 Dec 13;24(24):4580. doi: 10.3390/molecules24244580 (PMC6943482; doi:10.3390/molecules24244580)
Supplement: Supplementary file 1 [file molecules-24-04580-s001.pdf]

Table S1. Iodine absorbance spectrum parameters of starches from brown rice kernels

Rice Materials	OD620	OD550	OD620/550	λmax (nm)	Starch Component Changes
WT	0.188±0.004	0.210±0.009	0.89±0.02	568.2±1.0	
M005	0.157±0.012	0.203±0.018	0.78±0.01	553.2±2.4	+
M026	0.170±0.003	0.209±0.003	0.81±0.02	559.5±0.9	+
M028	0.184±0.002	0.213±0.001	0.86±0.02	565.3±1.1	
M030	0.189±0.004	0.211±0.000	0.89±0.02	569.1±3.0	
M031	0.102±0.007	0.148±0.012	0.69±0.01	542.7±2.5	+
M032	0.169±0.017	0.208±0.023	0.81±0.01	557.5±1.7	+
M038	0.188±0.006	0.222±0.011	0.85±0.02	561.3±1.1	
M041	0.162±0.003	0.188±0.002	0.86±0.01	567.4±0.8	+
M043	0.183±0.013	0.215±0.015	0.85±0.00	565.0±0.7	
M048	0.185±0.006	0.211±0.008	0.87±0.01	568.0±1.4	
M051	0.390±0.007	0.411±0.008	0.95±0.00	575.7±1.3	+
M063	0.181±0.007	0.199±0.008	0.91±0.00	571.8±0.4	
M068	0.170±0.003	0.207±0.007	0.82±0.01	558.5±1.3	+
M069	0.200±0.001	0.230±0.003	0.87±0.00	564.5±1.4	
M070	0.184±0.004	0.209±0.005	0.88±0.00	568.0±0.7	
M082	0.173±0.001	0.195±0.003	0.88±0.01	567.9±2.7	
M087	0.168±0.018	0.191±0.020	0.88±0.01	568.5±0.0	
M089	0.195±0.012	0.230±0.018	0.85±0.02	564.8±1.8	
M098	0.198±0.003	0.215±0.005	0.92±0.01	572.0±2.1	
M099	0.145±0.013	0.185±0.014	0.78±0.01	555.4±0.6	+
M103	0.169±0.009	0.206±0.008	0.82±0.01	560.3±0.4	+
M104	0.194±0.003	0.224±0.004	0.87±0.00	565.3±1.1	
M105	0.180±0.004	0.213±0.007	0.85±0.01	561.3±0.3	
M106	0.190±0.007	0.223±0.003	0.85±0.02	561.5±1.4	
M107	0.209±0.015	0.243±0.015	0.86±0.01	566.2±1.8	+
M108	0.206±0.004	0.243±0.006	0.85±0.00	561.5±0.0	
M109	0.187±0.007	0.221±0.006	0.85±0.01	564.0±0.0	
M110	0.195±0.008	0.230±0.008	0.85±0.01	561.3±0.4	
M111	0.178±0.006	0.194±0.011	0.92±0.02	573.0±0.7	
M112	0.161±0.002	0.190±0.004	0.84±0.01	562.7±1.7	+
M113	0.203±0.013	0.229±0.013	0.89±0.01	566.8±0.3	
M114	0.205±0.006	0.236±0.004	0.87±0.01	564.2±1.7	
M116	0.202±0.004	0.237±0.001	0.85±0.01	563.5±0.7	
M117	0.169±0.003	0.213±0.004	0.79±0.00	557.7±1.7	+
M119	0.186±0.001	0.219±0.002	0.85±0.00	564.0±2.1	
M120	0.196±0.002	0.231±0.003	0.85±0.00	565.0±0.7	
M122	0.152±0.008	0.200±0.008	0.76±0.01	553.5±0.5	+
M124	0.195±0.006	0.230±0.011	0.85±0.02	563.5±2.1	
M125	0.196±0.001	0.231±0.002	0.85±0.01	561.5±2.1	
M126	0.154±0.003	0.202±0.003	0.76±0.00	552.8±0.6	+

M129	0.185±0.006	0.217±0.004	0.85±0.01	561.5±1.5	
M130	0.195±0.008	0.230±0.013	0.85±0.01	561.7±1.8	
M131	0.088±0.008	0.145±0.013	0.61±0.00	533.3±0.3	+
M134	0.197±0.011	0.229±0.014	0.86±0.01	565.3±0.3	
M135	0.183±0.017	0.227±0.020	0.81±0.01	559.4±0.6	+
M136	0.207±0.006	0.235±0.001	0.88±0.02	568.1±3.6	
M138	0.202±0.006	0.234±0.011	0.86±0.01	565.3±1.1	
M147	0.200±0.007	0.236±0.008	0.85±0.00	564.3±1.0	
M148	0.207±0.001	0.244±0.001	0.85±0.00	563.0±0.0	
M149	0.148±0.006	0.181±0.006	0.82±0.01	566.0±2.1	+
M150	0.200±0.027	0.235±0.031	0.85±0.00	562.5±0.1	
M152	0.203±0.012	0.230±0.015	0.88±0.00	567.3±1.0	
M156	0.208±0.004	0.244±0.004	0.85±0.00	563.3±1.1	
M159	0.199±0.006	0.231±0.005	0.86±0.01	566.8±3.2	
M160	0.071±0.006	0.112±0.009	0.63±0.00	529.8±0.7	+
M163	0.186±0.005	0.220±0.005	0.85±0.00	563.0±0.7	
M164	0.178±0.004	0.210±0.004	0.85±0.01	561.2±0.3	
M165	0.127±0.005	0.169±0.007	0.75±0.01	549.9±0.3	+
M166	0.186±0.004	0.219±0.004	0.85±0.00	561.8±1.8	
M168	0.193±0.002	0.221±0.002	0.87±0.00	561.5±0.7	
M169	0.202±0.012	0.233±0.013	0.87±0.00	566.5±0.7	
M170	0.206±0.000	0.238±0.000	0.87±0.00	566.3±0.3	
M174	0.197±0.013	0.230±0.013	0.86±0.01	565.0±2.1	
M175	0.170±0.008	0.200±0.010	0.85±0.00	563.8±1.1	
M177	0.193±0.012	0.226±0.015	0.85±0.00	564.5±0.7	
M181	0.197±0.008	0.232±0.008	0.85±0.01	563.8±0.4	
M183	0.178±0.008	0.219±0.011	0.81±0.00	559.0±1.5	+
M188	0.186±0.004	0.220±0.001	0.85±0.02	561.8±1.1	
M193	0.185±0.002	0.218±0.001	0.85±0.01	559.0±0.7	
M196	0.175±0.004	0.226±0.010	0.77±0.02	553.8±0.8	+
M197	0.153±0.005	0.197±0.011	0.78±0.02	555.5±0.9	+
M204	0.177±0.007	0.184±0.011	0.96±0.02	574.0±0.5	+
M205	0.188±0.001	0.208±0.002	0.91±0.00	572.0±1.5	
M206	0.178±0.006	0.199±0.006	0.89±0.00	568.3±2.5	
M207	0.186±0.001	0.213±0.002	0.87±0.01	566.5±0.1	
M208	0.200±0.008	0.233±0.009	0.86±0.00	565.0±2.1	
M209	0.186±0.000	0.206±0.001	0.90±0.01	570.8±1.0	
M210	0.181±0.017	0.228±0.023	0.79±0.01	555.7±0.6	+
M212	0.200±0.010	0.235±0.006	0.85±0.06	561.7±6.0	
M217	0.172±0.004	0.202±0.003	0.85±0.01	563.3±0.4	
M219	0.168±0.009	0.213±0.010	0.79±0.01	557.8±1.0	+
M220	0.207±0.006	0.236±0.006	0.88±0.00	567.8±1.8	
M222	0.191±0.002	0.226±0.000	0.85±0.01	561.4±0.5	
M223	0.207±0.011	0.244±0.009	0.85±0.01	567.3±0.3	

M226	0.082±0.009	0.137±0.015	0.60±0.00	529.5±1.8	+
M227	0.145±0.007	0.182±0.010	0.80±0.01	557.6±4.0	+
M228	0.192±0.006	0.226±0.008	0.85±0.00	562.7±0.4	
M231	0.088±0.007	0.135±0.011	0.65±0.00	536.2±1.3	+
M235	0.157±0.008	0.200±0.007	0.78±0.01	556.2±1.4	+
M236	0.191±0.001	0.221±0.005	0.87±0.01	563.8±1.1	
M239	0.170±0.004	0.200±0.007	0.85±0.01	562.8±0.3	
M246	0.170±0.001	0.200±0.001	0.85±0.01	563.0±0.6	
M247	0.198±0.004	0.233±0.005	0.85±0.00	562.8±0.4	
M248	0.199±0.007	0.233±0.007	0.85±0.01	561.5±0.8	
M249	0.196±0.001	0.231±0.005	0.85±0.02	562.0±2.9	
M250	0.198±0.003	0.233±0.001	0.85±0.01	562.8±0.4	
M251	0.207±0.002	0.237±0.002	0.87±0.00	566.5±0.7	
M252	0.107±0.003	0.162±0.006	0.66±0.01	540.4±0.5	+
M253	0.188±0.012	0.231±0.017	0.81±0.01	560.0±0.5	+
M254	0.190±0.010	0.224±0.013	0.85±0.01	563.2±1.0	
M255	0.082±0.006	0.129±0.012	0.63±0.01	531.8±0.2	+
M263	0.205±0.005	0.233±0.007	0.88±0.01	567.3±1.0	
M265	0.197±0.003	0.229±0.007	0.86±0.01	565.0±1.4	
M266	0.195±0.009	0.202±0.010	0.97±0.01	579.7±1.9	+
M268	0.207±0.001	0.237±0.005	0.87±0.02	564.8±1.0	
M270	0.182±0.011	0.224±0.015	0.82±0.01	560.5±0.5	+
M272	0.198±0.008	0.233±0.013	0.85±0.01	561.8±1.8	
M273	0.200±0.005	0.236±0.009	0.85±0.01	563.5±1.5	
M274	0.183±0.002	0.219±0.003	0.84±0.01	562.4±1.1	+
M278	0.186±0.004	0.219±0.007	0.85±0.01	562.0±0.0	
M279	0.088±0.012	0.143±0.018	0.62±0.01	536.4±1.3	+
M282	0.173±0.000	0.195±0.000	0.89±0.00	567.8±4.6	
M283	0.185±0.006	0.218±0.004	0.85±0.02	561.5±1.5	
M284	0.202±0.004	0.238±0.002	0.85±0.02	563.5±0.7	
M286	0.207±0.004	0.241±0.005	0.86±0.00	564.0±0.8	
M288	0.191±0.004	0.226±0.008	0.85±0.01	564.3±0.4	
M290	0.157±0.007	0.197±0.013	0.80±0.02	556.0±1.3	+
M291	0.177±0.014	0.225±0.019	0.79±0.00	555.3±0.3	+
M294	0.207±0.011	0.243±0.010	0.85±0.01	564.3±1.0	
M295	0.204±0.000	0.236±0.005	0.87±0.02	566.5±3.6	
M296	0.201±0.007	0.237±0.008	0.85±0.00	562.0±1.5	
M299	0.176±0.000	0.205±0.002	0.86±0.01	565.0±0.7	
M301	0.170±0.008	0.214±0.012	0.80±0.01	557.2±0.8	+
M302	0.083±0.006	0.125±0.010	0.66±0.01	545.5±8.2	+
M303	0.202±0.010	0.232±0.006	0.87±0.02	567.2±2.4	
M307	0.201±0.006	0.236±0.008	0.85±0.00	564.3±1.1	
M320	0.189±0.007	0.222±0.010	0.85±0.01	562.1±0.1	
M333	0.092±0.009	0.141±0.010	0.65±0.01	535.0±0.9	+

M360	0.184±0.005	0.217±0.006	0.85±0.00	562.0±2.1	
M363	0.168±0.009	0.195±0.008	0.86±0.01	565.5±2.1	
M459	0.177±0.005	0.209±0.008	0.85±0.01	562.8±3.2	
M464	0.182±0.005	0.219±0.007	0.83±0.02	562.5±2.0	+
M477	0.169±0.013	0.194±0.012	0.87±0.01	567.8±2.4	
M482	0.163±0.001	0.206±0.005	0.79±0.02	558.9±2.6	+
M493	0.219±0.007	0.224±0.010	0.98±0.02	579.5±3.5	+

Data are means ± SD, *n* = 3; WT: wild type rice cultivar Kitaake; M: mutant; +: mutant with altered starch component.
